# Which Factors in a Child Welfare Worker’s Environment Influence Their Decision-Making About Neglect? A Systematic Narrative Literature Review

**DOI:** 10.1177/15248380251320987

**Published:** 2025-03-12

**Authors:** Jennifer Gibbs, Barry Coughlan, Tessa Morgan, Arbenita Mikushnica, Samantha Phippard, Francesca Crozier-Roche, Taliah Drayak, David Graham, Jack Smith, Robbie Duschinsky

**Affiliations:** 1 University of Cambridge, Cambridge, UK; 2 Care Leavers’ Association, Manchester, UK

**Keywords:** child neglect, social work, thresholds, assessment, decision-making

## Abstract

This paper systematically reviews literature to date about how child welfare workers’ decisions about neglect are influenced by their environments. We conducted a systematic search of Web of Science, Embase, PsycInfo, Scopus, and Applied Social Sciences Index & Abstracts, looking for studies in English of any methodology, published since the 1989 Children Act, in which statutory thresholds in England are defined. We used child welfare workers’ definitions of neglect or, in the absence of this, defined “neglect” as harmful acts of omission by a caregiver (failure to do something necessary for a child’s well-being). We included the views of child welfare practitioners with or working toward a social work qualification and those completing statutory child protection investigations, whatever their qualifications. Thirty-nine studies met the criteria for review. Quality was assessed using the Mixed Methods Appraisal Tool and Gough’s Weight of Evidence framework. Themes were identified iteratively and synthesized using a narrative method. The review found evidence that high caseloads and work-related stress might result in neglect receiving lower priority for intervention. The threshold for statutory intervention also appears to be influenced by the availability of non-statutory support services. These findings suggest that greater investment in services, particularly early intervention, may help children experiencing neglect receive sufficient, timely, and proportionate support. We also found that practitioners find it challenging to evidence neglect, and this can result in delays intervening, suggesting that practitioners may benefit from training and support in court skills.

## Introduction

In both the United Kingdom (Department for Education, 2024) and the United States (U.S. Department of Health & Human Services, 2023), neglect is the most commonly documented form of child maltreatment. Definitions of child neglect generally refer to “acts of omission”, that is, the failure of a person with parental responsibility to do something necessary for a child’s well-being, such as providing inadequate supervision and failing to meet children’s physical, emotional, and educational needs (Child Welfare Information Gateway, 2018, 2022, p. 3; HM Government, 2023, p. 160). In extreme cases, neglect may lead to death or serious injury (Brandon, 2013; Dickens et al., 2022). It is also associated with a range of long-term psychological difficulties (Coughlan et al., preprint).

Child welfare workers, including qualified social workers, play a pivotal role in responding to reports of child neglect. They assess risk and oversee interventions, including applying to court for a child to be removed from their parents’ care (HM Government, 2023). However, working definitions of neglect have been shown to be complex, multi-faceted, and somewhat subjective (Coope & Theobald, 2006; Horwath, 2001; Stone, 1998). Recent research has highlighted a dearth of evidence-based tools for the measurement of neglect (Duman et al., 2024; Haworth, Schaub, Kidney, et al., 2024). Practitioners report uncertainty about thresholds for intervention (Casey & Hackett, 2021; Chaudhry, 2016; Horwath, 2001), and there is evidence of level of inconsistency in decision-making between child welfare workers, even when standardized assessment tools are used (Arruabarrena & De Paúl, 2012; Ashton, 1999; Berrick et al., 2020; Bolton et al., 2021). This variation likely reflects how, as demonstrates, the “idea” of child abuse and neglect, distinct from the “reality” of children’s experiences, is not fixed but is constructed in a social “matrix.” These conceptions, beliefs, theories, and attitudes are shared through institutional norms and conventions. Literature about decision-making increasingly recognizes the influence of these social factors (e.g., Dickens et al., 2023), with the application of systemic (Munro, 2011) and ecological (Baumann, 2011; Benbenishty & Davison-Arad, 2012) models (Platt & Turney, 2013). Understanding factors which influence decision-making is essential to improving practice and policy design.

Several factors in a child welfare worker’s environment have the potential to influence their responses to neglect. First, there is the orientation of national policy and service design. Definitions of need and risk have broadened since the mid-1990s, leading to contact with more families (Gilbert et al., 2011; Institute for Government, 2023; USA Facts, 2020). Gilbert et al. (2011) have identified three broad approaches to service design: what they term a “child protection” orientation, where public services are targeted at families at high risk of abuse or neglect; a “family services” model, offering universal, preventative interventions, and a “child” orientation focusing on children’s rights. The orientation of services may vary over time and between countries according to political culture and resources. Demand for services also influences thresholds (Hood et al., 2019, 2020; Seppälä et al., 2024). For example, in England, the Children Act (Children Act, 1989) created a hybrid “family support” and “child protection” model. However, in 2010/11 to 2020/21, state funding for early intervention reduced by £1.9 billion (Larkham, 2024; Pro Bono Economics, 2022), resulting in a shift in practice toward the “child protection” orientation (Berrick et al., 2020) and later intervention (Larkham, 2024). The MacAlister review has recently recommended shifting back toward a greater emphasis on “family services” (MacAlister, 2022), with a similar focus on prevention in recent policy developments in the United States (Family First Prevention Services Act, 2018).

A second structural factor that has the potential to influence practice is working conditions. There is growing recognition that child welfare workers experience extremely high levels of occupational stress in comparison to other professions (McFadden et al., 2015; Ravalier et al., 2021, 2022). This leads to psychological distress (McFadden et al., 2015) and difficulty retaining experienced staff (Department for Education, 2023; Healy et al., 2009). These pressures may influence child welfare workers’ capacity to respond effectively where there are concerns about child neglect.

Third, it is important to recognize the role of the family courts, without whom a child welfare workers cannot intervene in a family’s life against their will. Some authors have alleged that a “culture of diffidence” (Stevenson, 1996, p. 15) exists, with child welfare workers finding legal action for neglect “too daunting” (Minty & Pattinson, 1994, p. 734). These papers are discursive in nature and are now quite dated. It would, therefore, be interesting to see whether this view is supported by contemporary empirical data.

Previous reviews on the topic of neglect have tended to concentrate on the causes and consequences of neglect (e.g., Tanner & Turney, 2003). Gibbs et al. (2024) have recently reviewed factors relating to the family, such as parental substance misuse and poverty, which influence practitioners’ assessments of neglect. However, it did not consider the role of factors in the decision-maker’s environment. We are aware of only one review that has considered environmental influences (Chaudhry, 2016), and this did not specifically demonstrate their impact on decision-making. It also did not include quality appraisal or an explicit method of synthesis and was unpublished.

## Aim

The aim of this paper was to identify what is known about how a child welfare workers’ environment influences their decision-making about neglect.

## Method

### Search Strategy

To answer this question, a systematic review of the literature was conducted according to PRISMA guidelines (Page et al., 2021) (see Supplemental Appendix 1 for PRISMA checklist). We included empirical studies of any methodology. As we approached this study with the understanding that definitions of neglect are socially constructed, we accepted the definitions of neglect given by the child welfare workers or researchers in the selected studies. Where studies reported responses to case vignettes without stating which type of maltreatment they represented, we classified as “neglect” those vignettes featuring harmful acts of omission by a caregiver (failure to do something necessary for a child’s well-being), as per the U.S. and U.K. definitions (Child Welfare Information Gateway, 2022, p. 3; HM Government, 2023, p. 160), and excluded acts of commission (proactively doing something harmful to the child). As found in other reviews about neglect (e.g., Taylor et al., 2012), articles frequently conflated findings about neglect and other forms of abuse. To ensure relevance, only findings specifically about neglect were included. “Child welfare workers” were defined as child welfare practitioners with responsibility for statutory responses to concerns about neglect. Within this, we included those with or working toward a social work qualification and those with non-social work qualifications.

Web of Science, Embase, PsycInfo, Scopus, and Applied Social Sciences Index and Abstracts were searched between July 17 and 29, 2021 and updated on October 28, 2024 (see Supplemental Appendix 2 for search strategy). Filters identified articles in English written after the passing of the Children Act (1989) in England, in which current thresholds are defined. The search yielded 2555 studies once duplicates (*k* = 2,040) were removed (see Supplemental Appendix 3). We then looked at these papers’ reference lists to identify an additional 37 relevant studies, including one item of “grey literature” (Horwath, 2001) containing supplementary data from two published studies (Horwath, 2004, 2005).

**Figure 1.**
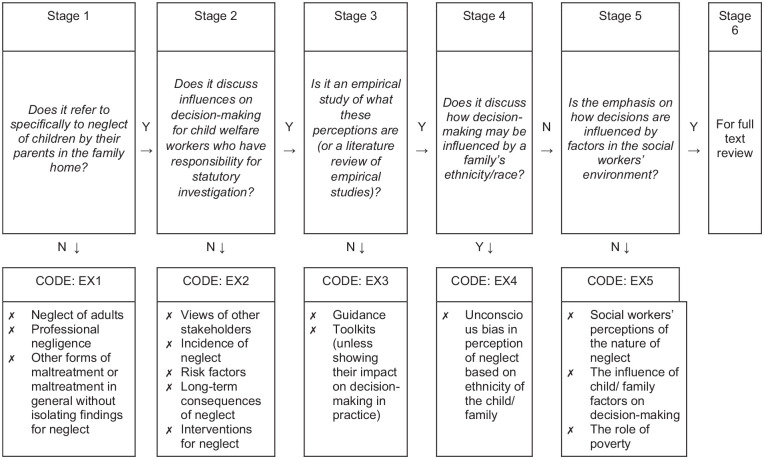
Title and abstract screening decision flowchart.

Inclusion criteria were then refined, deciding to also exclude material exploring how racial stereotypes influence decision-making. While this is unquestionably an important issue, we felt that this was a topic in its own right, and there was insufficient space to do it justice here. Table 1 states the final inclusion and exclusion criteria. Using Rayyan, J.G. screened all titles and abstracts (Figure 1), identifying 126 potentially relevant articles. A.M. independently screened 10% of these and, following discussion and clarification of criteria, there was complete agreement. J.G. then extracted and screened full texts.

**Table 1.** Critical Findings.

Contextual Influences on Child Welfare Workers’ Perceptions of Child Neglect	
The individual social worker	• Child welfare workers use their own experiences as a point of reference. • The impact of age and gender is uncertain. • Workers with child protection experience may have higher thresholds for intervention.
The organization	• Thresholds for intervention may rise if support services are available. • When caseloads and stress levels are high, thresholds for intervention may increase. • Compliance with timescales may be prioritized over quality in assessments.
The legal arena	• Child welfare workers’ and family court judges’ thresholds are similar. • Child welfare workers feel anxious about meeting the standard of evidence required in court for cases concerning chronic neglect. • Physical neglect may be prioritized over emotional neglect as it is easier to evidence. • Empowered parents may be able to challenge evidence about neglect.
National policy	• National policy influences child welfare workers’ perceptions of what neglect is and what interventions are appropriate. • Child welfare workers working in countries with a “child protection” policy orientation, where only families where there are significant levels of concern are targeted for state interference, had higher thresholds. • Child welfare workers’ discourse reflected their country’s political culture in terms of the balance between individual and societal responsibility for neglect. • Thresholds for intervention reduced following high-profile cases in the media concerning neglect.

### Data Extraction

Evidence was extracted by logging study characteristics (e.g., sample size, demographics, and methodology) on a table (Popay, 2006). Themes were identified iteratively, adding a column onto the table for each new theme as they were created. Findings were plotted into these columns, with quantitative data translated into themes.

### Quality Appraisal

Gough’s Weight of Evidence framework (Gough, 2007) was used to classify studies as being of high, medium, or low quality in relation to this review’s question (see Supplemental Appendix 4). This framework was chosen as it is suitable for various methodologies and accommodates the fact that some studies asked a different research question to this review. To give additional rigor to the first element of Gough’s framework, studies’ quality in their own right, J.G. applied the Mixed Methods Appraisal Tool (Hong QN et al., 2018).

### Analysis and Evidence Synthesis

These findings were then synthesized using an adapted form of Popay (2006)’s narrative method (see Supplemental Appendix 5). J.G. reflected on her own positionality as a practicing social worker through supportive discussions with other social workers (A.M., S.P.), academics (B.C., T.M., R.D.) and experts by experience (F.C.-R., T.D., D.G., and J.S.).

## Results

Thirty-nine studies met our full inclusion criteria, of which 35 included discrete samples. The majority (*k* = 36) were from peer-reviewed journals; two were theses and one a local government report. Most of the studies were conducted in the United States (*k* = 12), United Kingdom (*k* = 16), or Ireland (*k* = 3). Sixteen were assessed as being of high quality and 23 as medium quality. Where stated, 71% to 88% of respondents were female, which is comparable to the profession as a whole (87% in the United Kingdom (Department for Education, 2023), 81% in the United States (Deloitte, 2017)).

We identified two types of question asked by the selected studies (a) factors which child welfare workers thought influenced their decisions in a hypothetical situation and (b) factors which influenced child welfare workers’ decisions in actual practice, potentially unconsciously. Among studies of hypothetical situations, 17 studies used interviews or focus groups and 15 used surveys. Studies of decision-making in practice comprised nine examinations of case files, five statistical analyses of administrative records, and four ethnographic or observational studies. Some studies used more than method to provide evidence in both categories. The main themes we identified were characteristics relating to the individual child welfare worker, the organization, the legal threshold, and national policy.

## The Individual Child Welfare Worker

The first theme examines factors relating to the individual child welfare worker, where the evidence was relatively scarce and inconclusive.

### Practitioner Characteristics, Such as Age, Cultural Identity, and Attitudes, May Influence Decision-Making About Neglect

The suggestion that decisions about neglect may be influenced by personal characteristics featured as a theme in three qualitative studies (Grégoire-Labrecque et al., 2020; Horwath, 2004; Tufford et al., 2015). Several quantitative studies explored the nature of the relationship by measuring workers’ responses to hypothetical case vignettes. Two of these, one of which was high-quality, explored an association between a worker’s age and their decisions. Findings were contradictory: one found no association (Freisthler et al., 2017); the other found that older child welfare workers were more likely to decide that a child was at risk of neglect (Proctor, 2012). One explanation of the difference could be differences in vignette composition (the former featured parental substance misuse, the latter parental intellectual disability), although the mechanisms underlying this difference are unclear. Only one study, which was deemed of high quality, explored the role of gender and found no association with decision-making about neglect arising from parental substance misuse (Freisthler et al., 2017). Two studies, one of which was high-quality, explored the significance of a child welfare worker’s cultural heritage. Child welfare workers in the United States considered vignettes relating to physical neglect for example, medical and nutritional neglect, to be the most serious, while social workers in India were most concerned about neglect arising from parental substance misuse (Segal, 1992). Segal suggests that this reflected differences in living standards. However, cultural values may also have been relevant since another study showed that, within one country (the United States), social workers of white ethnicity were the least concerned about substance misuse (Freisthler et al., 2017). Finally, one high-quality study explored the relevance of a worker’s psychology, finding that decisions were influenced by their emotions, experiences, values, and social norms (Rodrigues et al., 2015).

### Child Welfare Workers With More Experience in Statutory Roles May Have Higher Thresholds for Intervention

Six studies (seven papers), three of which were high-quality, considered how experience and education influenced child welfare workers’ decision-making. Three quantitative studies that analyzed responses to case vignettes presented a mixed picture. One found no statistically significant association between levels of experience and decision-making (Coohey, 2003), whereas two studies found that workers with more experience in child welfare settings tended to have higher thresholds (Denne et al., 2019; Freisthler et al., 2017). The latter finding was also supported by a qualitative study, where one of the social workers interviewed commented that: “newly qualified workers . . . have a case of neglect and feel that it needs the children perhaps removing . . . a more experienced worker may go in there and say . . . the threshold isn’t quite met” (Casey & Hackett, 2021, p. 2106). More studies, therefore, suggested that experience could influence decision-making, leading to higher thresholds, but the evidence is scarce and is therefore inconclusive.

Turning next to job role, one study suggested that staff in investigatory roles rated neglect vignettes more seriously than those in service roles, although the difference was only statistically significant for vignettes concerning emotional neglect (Rose & Meezan, 1995, 1996). Initially, this would appear to contradict the former studies, suggesting that those with more child protection experience had lower thresholds. However, Rose and Meezan note that, at the time, the state studied allowed individuals without a social work qualification to work in investigatory child welfare roles, for example, police, who have a different training route and value base, which may explain the difference.

Only one survey study considered the influence of education on caseworkers’ decision-making about neglect. This found that those with relatively low (less than a Bachelor’s degree) or relatively high (more than a Master’s degree) levels of education were more likely to perceive that a vignette featuring parental substance misuse amounted to neglect (Freisthler et al., 2017). However, while the study controlled for current job role and age, it did not control for levels of experience so some confounding may be present. One paper showed how a child welfare worker’s knowledge of specific issues (in this case, pediatric burns) could also influence their decision-making (Cowley et al., 2024).

## The Organization

The second group of themes concerned organizational influences on child welfare workers’ decision-making about neglect.

### Child Welfare Workers’ Thresholds About Neglect May be Influenced by Available Resources

Four studies, all of medium-quality, explored the impact of resource availability. Three studies, including studies of both hypothetical and actual decision-making, suggested that child welfare workers were less likely to recommend that a child be removed from their parents’ care if alternative support services were available (Agastya et al., 2024; King et al., 2021; Rossi et al., 1999). One study presented a potentially contrasting picture. Here, social workers surveyed thought that neglect allegations would be more likely to receive ongoing involvement in urban communities (Craft & Staudt, 1991) due to differing workloads and community norms. However, the authors do not clarify whether resources were perceived to be more or less abundant in the urban setting, so it is not possible to decide if it represents a challenge to the former evidence. This study also only reports what child welfare workers thought would happen, not what actually happened, and is therefore of lower quality. Thus, it appears likely that the more support services are available, the higher thresholds for statutory intervention, but the research is too scarce to be conclusive.

### Stress and Workload May Affect Decisions About Neglect

Nine studies, three of which were high-quality, explored how stress influenced decision-making about neglect. Of these, six asked child welfare practitioners about the impact of stress on their decisions in the abstract. These presented consistent findings that the quality of assessments and interventions may be compromised when child welfare workers experience high levels of stress (Chaudhry, 2016; Daniel et al., 2014; Dickens et al., 2023; Haworth, Schaub, & Montgomery, 2024; Horwath, 2001). One social worker interviewed explained that high workloads meant that they, “only have time to take the most in need” (Casey & Hackett, 2021, p. 2105) and that “borderline” neglect referrals would get declined and re-referred several times before any action was taken (Casey & Hackett, 2021, p. 2105).

Studies of decision-making in practice were also in agreement with those asking about hypothetical concerns, with evidence that better-resourced teams exercised lower thresholds for intervention and assessed children’s needs more holistically (Horwath, 2004). Two qualitative studies explored the mechanisms of this, finding evidence that child welfare workers became “desensitized” (Casey & Hackett, 2021) and started to “normalize” (Taylor et al., 2024) neglect. An observational study also found that when student social workers were overwhelmed by a mother’s vulnerability, they were at risk of overlooking the harm to her baby (Tufford et al., 2015). Meanwhile, Denne et al (2019) identified compassion fatigue as a moderating factor in the association between a workers’ level of experience and their decision-making discussed above. Thus, a number of studies consistently suggested that stress influences child welfare workers’ decision-making, leading to higher thresholds for intervention.

### Child Welfare Workers’ Decisions May be Influenced by Assessment Tools

Only three of the selected studies, two of which were high-quality, explored how assessment tools influenced decision-making about neglect in practice. Referrals for neglect were more likely to be transferred for ongoing support, relative to other forms of maltreatment, when social workers used a tool that emphasized future risk (King et al., 2021). Nonetheless, tools were not always used as intended, with some sections, such as that on environmental factors, only partially completed (Casey & Hackett, 2021). Casey and Hackett suggested that this might be due to a lack of training, but another study found that training did not eliminate inconsistent practice (Arruabarrena & De Paúl, 2012). Meanwhile, in interviews, some social workers commented that they disliked standardization, feeling that this impeded a holistic, analytic view of neglect (Casey & Hackett, 2021, p. 2107). Conclusions about this theme are, therefore, unclear due to the scarcity of evidence.

### Team Culture May Influence Decision-Making About Thresholds

Only three papers, two of which from the same study and all high-quality, explored the role of team culture. Observations of practice noted that definitions of neglect could vary slightly between different teams and supervisors (Horwath, 2004, 2005). In interviews, workers also complained that a performance-management culture led to compliance with timescales being prioritized over quality in assessments about neglect (Casey & Hackett, 2021). Again, the evidence is too limited to fully understand this area.

## The Legal Context

### Child Welfare Workers May Feel Anxious About Meeting the Legal Standard of Evidence for Neglect Cases

#### This Might Influence Their Decisions About Different Forms of Intervention

Ten studies, seven of which were high-quality, gave insights into how child welfare workers’ were mindful of the need to justify their decisions about neglect against the legal threshold for intervention. First, several studies suggested that emotional neglect received lower priority for intervention at all levels because it was harder to evidence. An ethnographic study found that practitioners paid greater attention to physical needs, partly due to the “usefulness of the observable body as evidence,” as “concrete proof” (Scourfield, 2000, pp. 377–378). Similarly, analysis of case files found an emphasis on forms of neglect for which there were “tangible and easily measurable” outcomes (Horwath & Tarr, 2015, p. 1389) such as attending school or appointments. One study interviewed workers about their work with affluent families, where emotional harm is the most common reason for contact with child welfare services. This showed how practitioners’ claims that emotional neglect had occurred were subject to challenge by lawyers or powerful contacts, sometimes prompting child welfare workers to change their decisions (Bernard, 2019; Bernard & Greenwood, 2019).

As well as influencing the focus of interventions, the selected studies provided some evidence that concerns about evidencing neglect informed workers’ decision about when to apply to the family courts to ask for a child to be placed in out-of-home care. One survey study compared responses to neglect vignettes from social workers and family court judges and found their decision-making to be similar, suggesting a level of mutual influence (Berrick et al., 2020). However, a more complex relationship is revealed in qualitative studies. In three studies, interviewees suggested that it was difficult to persuade the courts that the threshold for action was met in cases of chronic neglect, meaning that sometimes applications were delayed until a more conclusive “catapult” (Dickens, 2007), “crisis” (Horwath, 2001), or “critical” (Doherty, 2017) event. Limited support was provided for this view from studies of actual practice. Administrative records showed that one-third of care proceedings for neglect were initiated following a “crisis” event (Farmer & Lutman, 2014) although the majority were not. Meanwhile, Lee (2016)’s observational study found that the family court “too often rubber-stamps caseworkers’ judgments with little scrutiny” (Lee, 2016, p. 140). This difference may be because Lee’s study was based largely on observations of court hearings, potentially only seeing cases which child welfare practitioners had pre-screened and judged to meet the court’s threshold. The available evidence is therefore sufficient to suggest that child welfare workers worry about intervening in neglect due to evidential challenges. Unfortunately, we found no evidence about why this was or what interventions might redress this.

## National Policy

### Child Welfare Workers’ Decision-Making About Neglect May be Influenced by National Policy and Political Culture

Six studies, of which three were high-quality, explored how national policy and political culture shape child welfare workers’ decision-making. First, there was evidence that child welfare workers were influenced by policy when deciding at what threshold they should intervene in a family’s life following concerns about neglect. Although not the focus of their paper, Berrick et al. (2020)’s raw data showed how responses to a case vignette concerning neglect varied between countries with what Gilbert et al (2011) classify as “family services” (universal) and “child protection” (targeted) orientations. Here, “family services” child welfare workers were more likely to decide that a vignette amounted to neglect and that a child should be removed (Berrick et al., 2020). Meanwhile, Janczewski (2015) and Janczewski & Mersky (2016) explored the impact of differential response on decisions in practice. This is a service design where referrals assessed as lower risk are diverted to non-statutory support services. The study showed that states using differential response had lower population-level rates of investigations and substantiation of neglect allegations, although some of the differences in substantiation rates predated the introduction of differential response. This finding is consistent with the information that decision-making is influenced by the availability of support services, discussed above.

We also found evidence that political culture influences child welfare workers’ decisions in terms of who they hold accountable for the neglect that has occurred. Two ethnographic studies found that, despite social work’s professed commitment to social justice, practitioners in the United Kingdom (Casey & Hackett, 2021) and the United States (Lee, 2016) demonstrated “neo-liberal” values in practice, emphasizing individual rather than societal failings in their decision-making (Casey & Hackett, 2021, p. 2104). However, one paper provided a contrast to this, giving insights into times when professional and personal standards took priority over official policy in shaping decisions. In this study, interviewees reported that when they found policy to be impractical or unfair, for example, demanding unaffordable levels of maintenance from fathers (Abdullah et al., 2021), they sometimes ignored it and practiced creatively outside of official guidelines. Possible explanations for the difference include contrasting political cultures, a more obvious link between poverty and neglect in a lower-income country or that Abdullah et al.’s study is based on practitioner self-reporting, which may be influenced by social desirability bias. There is, therefore, very limited information available about this theme. What is known indicates that policy and political culture may influence decisions about neglect but that other factors are also at work, such as pragmatism and professional values. Much more research is needed to understand the balance between these competing influences.

### Child Welfare Workers Decisions About Neglect May be Influenced by the Media

In addition to formal government policy, five studies, three of which were high-quality, found that media attention could influence decision-making about neglect. For example, two ethnographic studies observed how practice became more defensive following high profile child deaths (Lee, 2016; Scourfield, 2000). In interviews, social workers also mentioned that their decisions about thresholds for intervention were influenced by how child welfare was represented in the press (Casey & Hackett, 2021; Doherty, 2017) and inspection reports (Hood et al., 2020).

## Discussion

This is the first review to consider systematically how a child welfare worker’s environment influences their decisions about neglect. From this, it is clear that knowledge on this topic is very limited, with many themes only explored by a small number of studies.

The best available information is about how organizational issues, such as stress and resource shortages, affect decision-making. Although the studies are not numerous, the evidence so far is consistent, showing that when child welfare workers operate in pressured environments, with high caseloads and levels of stress, the threshold at which child welfare workers decide to offer an intervention to children experiencing neglect may rise. There was also limited evidence that when organizations and national policy do not provide the option of non-statutory support services, child welfare workers are more likely to recommend a statutory response. These findings about factors influencing decision-making about neglect have parallels with research into decision-making about all forms of maltreatment collectively: high workloads lead to rising thresholds (All Party Parliamentary Group for Children, 2018; Font & Maguire-Jack, 2021; Graham et al., 2015; Hood et al., 2019; Lwin et al., 2024) and child welfare workers are less likely to make a decision of substantiation if the family can access alternative supports (Font & Maguire-Jack, 2015; Font & Maguire-Jack, 2021). However, our findings about neglect may be of particular concern, since Gibbs et al. (2024) have shown that neglect already tends to be ascribed lower priority for intervention than other forms of maltreatment. Font and Maguire-Jack (2021) have also shown that responses to neglect are influenced by organizational factors more than responses to sexual abuse. This paper, therefore, raises concerns that amid the pressures on frontline services outlined in the introduction, child welfare workers’ may struggle to provide children experiencing neglect with adequate, timely, and minimally intrusive support.

The available evidence also raised concerns that child welfare workers may find it hard to provide evidence that neglect, particularly emotional neglect, has occurred and that a particular level of intervention is justified. This suggests that the “culture of diffidence” child welfare workers feel toward the court arena (Stevenson, 1996, p. 15) has persisted. It also raised the concerning possibility that child welfare workers may delay intervention for neglect until a crisis incident occurs, meaning that children may suffer harm for longer.

Studies into the relevance of individual worker characteristics were scarce, with inconclusive results. Available information hints that a worker’s level of experience and attitudes may be more important than their demographic characteristics (age, gender). This would be in line with the theme about stress leading to desensitization. It would also be consistent with what is known about the impact of worker characteristics on decision-making about maltreatment in general, which have found no association between decision-making age and gender (Abner, 1997; Ashton, 2004) but some association with levels of experience (Abner, 1997) and attitudes toward removing a child (Arad-Davidzon & Benbenishty, 2008; Davidson-Arad & Benbenishty, 2010). Other themes were present in the literature, but were represented by only a few studies, meaning that it is difficult to draw any firm inferences.

### Limitations of Current Knowledge

The review has found that this topic is under-researched generally and that further studies on all themes identified would be beneficial. Nonetheless, a number of particular gaps were identified. The first gap is about how child welfare worker variables influence decision-making. The availability of information was reduced by the fact that several survey studies did not differentiate between different forms of maltreatment when reporting survey responses and therefore had to be excluded. Most evidence was about how diversity influences decisions about hypothetical case vignettes, not actual practice decisions, meaning that it is unknown if effects are moderated by other factors, such as the chain of command. There was also no evidence about the influence of some demographic characteristics, for example, gender identity, sexual orientation, and disability, while ethnicity/culture was considered crudely, not considering cultural integration (e.g., for a third generation immigrant). Included survey studies did not always adequately control for confounding or moderating variables, for example, one measured age but not related factors such as parental status (Proctor, 2012). Sometimes the response rate was unknown (Freisthler et al., 2017; Proctor, 2012). A final issue was that two of the survey studies only explored decision-making about neglect in relation to very specific sub-groups, parents with an intellectual disability (Proctor, 2012) or parents who misused substances (Freisthler et al., 2017), which may limit generalizability to other settings. There were also issues with the quality of reporting. All the survey studies based their analysis on null-hypothesis statistical testing (NHST) rather than confidence intervals. NHST has faced criticism for being difficult to interpret and encouraging potentially misleading dichotomous conclusions (Cumming, 2014). Furthermore, NHST was not always well executed. Sample sizes were relatively small, for example, 123 (Rose & Meezan, 1995, 1996) and 230 (Proctor, 2012), creating very small subgroups and few justified their sample size, meaning that it is unknown whether the sample was large enough to detect differences if they existed in the population. Finally, the studies used different instruments, prohibiting meta-analysis. Findings concerning the theme of child welfare worker demographics therefore need to be read with caution. This said, demographic factors have been found to be less important than case characteristics in predicting decisions about maltreatment overall (Abner, 1997; Ashton, 1999; Freisthler et al., 2017; Lwin et al., 2024; Rose & Meezan, 1995, 1996; Rossi et al., 1999; Segal, 1992). There is also evidence that the impact of workers’ attitudes on actual decisions may be moderated by training and professional culture (Davidson-Arad & Benbenishty, 2014). Therefore, while there are clearly significant gaps in the evidence, this area may not be a high priority for future work.

The second limitation is about the geographical spread of studies to date. All information in English about the legal contexts and workloads comes from the United Kingdom, Ireland, and the United States. A third limitation is about changes in attitudes to neglect over time. None of the selected studies explored this specifically and differences in study instruments made it impossible to infer this by comparing different studies’ data. Fourth, no studies compared decisions with outcomes for children. Therefore, we can only infer rather than know which environments best enable child welfare workers to promote children’s well-being.

Finally, no information was found about a number of other factors which might also influence thresholds, such as training, supervision, IT systems, and interagency working. This may be due to study design in the quantitative papers. Nonetheless, these themes did not feature in the qualitative studies reviewed, including those using iterative methods of analysis, for example, narrative enquiry (Casey & Hackett, 2021) and grounded theory (Scourfield, 2000). This, therefore, gives confidence that the themes covered in this review were the most important to the child welfare workers who participated.

### Limitations of the Scope of the Review

The current review only searched academic databases, which is a limit of this review, particularly given how little social work research is published. Due to linguistic barriers, only items in English were included, leading to a geographical bias in the data. The review considered the influence of the family courts from a child welfare worker’s perspective but did not search legal databases so may have missed relevant material. Only one researcher assessed the quality of studies and their subsequent weight of evidence, creating some risk of bias, although this was moderated by reporting all study findings.

The requirement to exclude items not specifically about neglect means that the findings may not be applicable to cases of poly-victimization. Survey evidence suggests that 55% of the time, concerns are about more than one type of maltreatment (Euser et al., 2013), particularly for children with disabilities or children whose parents have long-term physical or learning disabilities or mental health issues (Radford et al., 2011). Focusing on neglect in isolation does, therefore, limit this review’s generalizability, particularly with these vulnerable groups.

## Conclusion

### For Practice

The scarcity of information identified on this topic means that any conclusions for practice must be tentative. The suggestion that stress and personal experiences may influence decision-making about neglect means that child welfare workers may benefit from reflective and supportive supervision to aid their decision-making. Unfortunately, we do not know why practitioners struggle to evidence that neglect has occured, making it difficult to suggest how confidence in this area may be improved. However, it is possible that training in how to demonstrate that harm caused by neglect justifies a particular intervention, perhaps making better use of research evidence, may be beneficial.

### For Policy and Service Design

Again, the weakness of existing evidence makes it difficult to make any firm recommendations for policy. However, tentative information suggests that resource pressures and insufficient provision of non-statutory support services may mean that children experiencing neglect either do not receive support at all or receive a more intrusive intervention than would otherwise have been necessary. The review, therefore, supports the case for greater investment and policy emphasis on early intervention, which has recently been enacted into legislation in the United States (Part IX: Guidelines for specific interventions, 2009) and is encouraged in a recent review of children’s social care in the United Kingdom (Daniel, 2015; MacAlister, 2022; see also Mckay, 2024).

### For Research

This review has found that a range of environmental factors have the potential to influence child welfare workers’ decision-making about neglect, although the extent of this is not fully understood due to limited information. Informant reporting, particularly from child welfare workers, is commonly used as an indicator of neglect (Stoltenborgh, Bakermans-Kranenburg et al., 2015). Caution is, therefore, needed when using child welfare worker substantiation to calculate incidence rates between areas or over time as there may be variation in the threshold as well as in the population. Second, there are implications when sampling substantiated reports of neglect for research purposes. If individual and organizational characteristics may create variety in thresholds for neglect, qualitative research may benefit from purposely sampling a diverse range of these characteristics. Meanwhile, quantitative studies may need to measure and control for these to ensure that they do not confound research into the impact of other variables.

**Table:** Implications for Practice, Policy, and Research.

Practice	• Reflective, supportive supervision may help child welfare workers recognize how their perceptions of neglect may be influenced by their personal circumstances and stress levels. • Child welfare workers would benefit from greater support and training in how to evidence the harm caused by neglect in the court arena.
Policy	• Failure to provide support services is likely to lead to increased involvement on a statutory basis for children experiencing neglect.
Research	• Caution is needed when using informant reporting as an indicator of neglect as thresholds are not entirely consistent. • For qualitative studies into neglect, generalizability may be improved by purposefully sampling cases from child welfare workers and organizations with a diverse range of characteristics. • For quantitative studies into neglect, the reliability of results may be improved by measuring and controlling for variables such as national policy and caseloads which influence thresholds for neglect.

## Supplemental Material

sj-docx-1-tva-10.1177_15248380251320987 – Supplemental material for Which Factors in a Child Welfare Worker’s Environment Influence Their Decision-Making About Neglect? A Systematic Narrative Literature Review Supplemental material, sj-docx-1-tva-10.1177_15248380251320987 for Which Factors in a Child Welfare Worker’s Environment Influence Their Decision-Making About Neglect? A Systematic Narrative Literature Review by Jennifer Gibbs, Barry Coughlan, Tessa Morgan, Arbenita Mikushnica, Samantha Phippard, Francesca Crozier-Roche, Taliah Drayak, David Graham, Jack Smith and Robbie Duschinsky in Trauma, Violence, & Abuse

sj-docx-2-tva-10.1177_15248380251320987 – Supplemental material for Which Factors in a Child Welfare Worker’s Environment Influence Their Decision-Making About Neglect? A Systematic Narrative Literature Review Supplemental material, sj-docx-2-tva-10.1177_15248380251320987 for Which Factors in a Child Welfare Worker’s Environment Influence Their Decision-Making About Neglect? A Systematic Narrative Literature Review by Jennifer Gibbs, Barry Coughlan, Tessa Morgan, Arbenita Mikushnica, Samantha Phippard, Francesca Crozier-Roche, Taliah Drayak, David Graham, Jack Smith and Robbie Duschinsky in Trauma, Violence, & Abuse

sj-docx-3-tva-10.1177_15248380251320987 – Supplemental material for Which Factors in a Child Welfare Worker’s Environment Influence Their Decision-Making About Neglect? A Systematic Narrative Literature Review Supplemental material, sj-docx-3-tva-10.1177_15248380251320987 for Which Factors in a Child Welfare Worker’s Environment Influence Their Decision-Making About Neglect? A Systematic Narrative Literature Review by Jennifer Gibbs, Barry Coughlan, Tessa Morgan, Arbenita Mikushnica, Samantha Phippard, Francesca Crozier-Roche, Taliah Drayak, David Graham, Jack Smith and Robbie Duschinsky in Trauma, Violence, & Abuse

sj-docx-4-tva-10.1177_15248380251320987 – Supplemental material for Which Factors in a Child Welfare Worker’s Environment Influence Their Decision-Making About Neglect? A Systematic Narrative Literature Review Supplemental material, sj-docx-4-tva-10.1177_15248380251320987 for Which Factors in a Child Welfare Worker’s Environment Influence Their Decision-Making About Neglect? A Systematic Narrative Literature Review by Jennifer Gibbs, Barry Coughlan, Tessa Morgan, Arbenita Mikushnica, Samantha Phippard, Francesca Crozier-Roche, Taliah Drayak, David Graham, Jack Smith and Robbie Duschinsky in Trauma, Violence, & Abuse

sj-docx-5-tva-10.1177_15248380251320987 – Supplemental material for Which Factors in a Child Welfare Worker’s Environment Influence Their Decision-Making About Neglect? A Systematic Narrative Literature Review Supplemental material, sj-docx-5-tva-10.1177_15248380251320987 for Which Factors in a Child Welfare Worker’s Environment Influence Their Decision-Making About Neglect? A Systematic Narrative Literature Review by Jennifer Gibbs, Barry Coughlan, Tessa Morgan, Arbenita Mikushnica, Samantha Phippard, Francesca Crozier-Roche, Taliah Drayak, David Graham, Jack Smith and Robbie Duschinsky in Trauma, Violence, & Abuse
