# Supplementary material for: Which Factors in a Child Welfare Worker’s Environment Influence Their Decision-Making About Neglect? A Systematic Narrative Literature Review
Source: Trauma Violence Abuse. 2025 Mar 12;27(3):611–23. doi: 10.1177/15248380251320987 (PMC13291384; doi:10.1177/15248380251320987)
Supplement: sj-docx-2-tva-10.1177_15248380251320987 – Supplemental material for Which Factors in a Child Welfare Worker’s Environment Influence Their Decision-Making About Neglect? A Systematic Narrative Literature Review [file sj-docx-2-tva-10.1177_15248380251320987.docx]

**Appendix 2: Search strategy**

Web of Science , Medline , Embase, PsychINFO, Scopus, Assia  were searched on 16-29th July 2021 and updated on 28^th^ October 2024.

Exclusion criteria within database:

- Articles published since 1989 (Children Act)
- English language

Search terms used in Medline (with appropriate adjustments for other databases):

"negl*" near/3 ("child*" or "infant" or "baby" or "adolesc*" or "youth" or "teen*" or “kid*” or “minor*” or “paediatr*” or “pediatr*” or “juven*” or “junior*” or “girl*” or “boy*” or “toddler*” or “pre-school*” or “young near/2 (adult* OR person* OR individual* OR people* OR population* OR man OR men OR wom#n) ”)

"percept*" or "defin*" or "threshold*" or "interpret*" or "understand*" or "perspective*" or "idea*" or  "identif*" or "concept*" or "view*" or "attitude*" or "opinion*" or "measure*" or "knowledge" or “assess*”

"children’s Service*" or "child service*" or "social service*" or "social Worker*" or "safeguarding" or "case worker*" or "CPS*" or "child Protection service*" or "child welfare service*"  Or MESH terms for Social Worker where available in database
