# Supplementary material for: Which Factors in a Child Welfare Worker’s Environment Influence Their Decision-Making About Neglect? A Systematic Narrative Literature Review
Source: Trauma Violence Abuse. 2025 Mar 12;27(3):611–23. doi: 10.1177/15248380251320987 (PMC13291384; doi:10.1177/15248380251320987)
Supplement: sj-docx-3-tva-10.1177_15248380251320987 – Supplemental material for Which Factors in a Child Welfare Worker’s Environment Influence Their Decision-Making About Neglect? A Systematic Narrative Literature Review [file sj-docx-3-tva-10.1177_15248380251320987.docx]

**Identification of studies via databases and registers**

Records identified from Web of Science, Medline, Embase, Psych INFO, Scopus, Assia

Databases (n = 6)

Registers (n = 0)

Databases = 4595

Snowballing = 37

Total = 4632

Records removed *before screening*:

Duplicate records removed (n =2040)

Records removed for other reasons (n = 17) (no abstract available)

**Identification**

Records screened.

(n = 2575)

Records excluded**

(n = 2449)

Reports sought for retrieval.

(n =126)

Reports not retrieved.

(n = 3)- full text not available

**Screening**

Reports excluded:

Reason 1 (n = 26)

Reason 2 (n = 8)

Reason 3 (n = 14)

Reason 4 (n = 5)

Reason 5 (n = 31)

Reports assessed for eligibility.

(n = 123)

Studies included in review.

(n = 39)

**Included**

*Consider, if feasible to do so, reporting the number of records identified from each database or register searched (rather than the total number across all databases/registers).

**If automation tools were used, indicate how many records were excluded by a human and how many were excluded by automation tools.

*From:*  Page MJ, McKenzie JE, Bossuyt PM, Boutron I, Hoffmann TC, Mulrow CD, et al. The PRISMA 2020 statement: an updated guideline for reporting systematic reviews. BMJ 2021;372:n71. doi: 10.1136/bmj.n71

For more information, visit: <http://www.prisma-statement.org/>
