# Supplementary material for: Which Factors in a Child Welfare Worker’s Environment Influence Their Decision-Making About Neglect? A Systematic Narrative Literature Review
Source: Trauma Violence Abuse. 2025 Mar 12;27(3):611–23. doi: 10.1177/15248380251320987 (PMC13291384; doi:10.1177/15248380251320987)
Supplement: sj-docx-4-tva-10.1177_15248380251320987 – Supplemental material for Which Factors in a Child Welfare Worker’s Environment Influence Their Decision-Making About Neglect? A Systematic Narrative Literature Review [file sj-docx-4-tva-10.1177_15248380251320987.docx]

**Appendix 4: Studies selected**

|  | Author | Title | Year | Country | Type of study | | | Subjects (n) | | | Research question | | | | | | Quality appraisal | | Relevance to this review | |  |
| --- | --- | --- | --- | --- | --- | --- | --- | --- | --- | --- | --- | --- | --- | --- | --- | --- | --- | --- | --- | --- | --- |
|  | Abdullah, A., Ayim, M., Bentum, H. and Emery, C. R. | Parental poverty, physical neglect and child welfare intervention Dilemma and constraints of child welfare workers in Ghana . | 2021 | Ghana | Semi-structured interviews. | | | 13 | | | To understand the dilemmas faced by social workers dealing with neglect in the context of poverty. | | | | | | A = H, B = M  C = M, D = M  Sampling described as “purposive” but had limited diversity (e.g. only recruited experienced workers). | | Social workers felt that existing state interventions (LEAP welfare assistance) were insufficient. Social workers sometimes refused to impose maintenance payments on fathers if they lacked the means to pay. | |  |
|  | Agastya, NLPM, Wise, S, Kertesz, M, Kusumaningrum, S. | Transformation of child welfare Institutions in Bandung, West Java: A case of deinstitutionalization in Indonesia | 2024 | Indonesia | Survey asking closed ended questions about the organisation that they worked for and then asked for responses to case vignettes. | | | 82 | | | To understand what services exist for children in Bandung who experience neglect. | | | | | | A = H, B = M, C = M, D = M  Detailed methodology. Mixed methods to provide different insights. Context of Indonesia is very different to the developed countries which are the topics of most of the other selected studies so potential to synthesise is limited. | | In response to a single vignette, some providers offered institutional care without family support, others were able to offer preventative services. Some workers operated in religious institutions, and this influenced their decisions (e.g. attitude to a child born outside of wedlock) | |  |
|  | Arruabarrena, I Ignacia and De Paúl, J. | Improving accuracy and consistency in child maltreatment severity assessment in child protection services in Spain: New set of criteria to help caseworkers in substantiation decisions. | 2012 | Spain | Survey asking participants to respond to case vignettes. | | | 515 caseworkers (half the workforce). | | | To see if the accuracy and consistency of assessments could be improved through training in a new assessment tool. | | | | | | A = H  B = H  C = M  D = H  Good sample size (515, half of the workforce) and response rate relative to other survey studies.  The study was primarily interested in the impact of training in a certain assessment tool and consistency with a “correct” “expert” opinion, whereas this review is interested in views more generally. | | Whilst training in the assessment tool improved consistency and accuracy (when compared to an “expert” opinion), significant inconsistency remained. | |  |
|  | Beck, K. and Vornanen, R. | Responding to Child Maltreatment: Comparison between School Social Work in Finland and Germany | 2019 | Finland and Germany | Surveys asking social workers to respond to a case vignette. | | | 8 | | | To use a comparative approach to identify areas in which practice could be improved. | | | | | | A = M, B = H  C = M, D = M  Very small sample, recruited via convenience methods. Vignette featured only 2 examples of neglect . | | Compared to social workers in Germany, school social workers in Finland were less concerned that a child was hungry as school meals are provided as standard. | |  |
|  | Bernard, C and Greenwood, T. | Recognizing and addressing child neglect in affluent families. | 2019 | England | 1. Interviews 2. Focus groups. | | | 30 | | | To better understand how neglect manifests and is perceived in affluent families. | | | | | | A = H, B = H  C = H, D = H  Diverse sample.  Explicit method of analysis (thematic analysis). | | Practitioners struggled to address neglect in affluent families. Concerns were most commonly about emotional neglect, which is harder to evidence. Parents also had powerful connections and utilised complaints procedures. | |  |
|  | Bernard, C. and Greenwood, T. | ‘We’re giving you the sack’—Social Workers’ Perspectives of Intervening in Affluent Families When There Are Concerns about Child Neglect. | | | | | | | | 2019 | | | | | This paper is based on the same study as the above paper and reports the same findings. | | | | | |  |
|  | Berrick, J., Dickens, J., Pösö, T. and Skivenes, M. | Are child protection workers and judges in alignment with citizens when considering interventions into a family? A cross-country study of four jurisdictions. | 2020 | England, USA (California), Norway, Finland | Survey asking workers to respond to statements about a case vignette using a Likert scale. Analysed using Mean, Standard Error, T-tests. | | | 1091 front line CP staff, 1691 judicial decision makers, 4003 members of the public | | | To compare social workers, judges, and members of the public’s views about neglect. | | | | | | A = M, B = H  C = M, D = M  Response rates unknown or relatively low (20-38%); no information about how representativeness of respondents. Only one vignette relating to neglect. No confidence intervals. | | Social workers from Norway, the “family services system” were most likely to agree that the vignette amounted to neglect and to recommend that a care order be made. Social workers from California and the USA, the “risk protection system” were the least likely. | |  |
|  | Casey, B and Hackett, S. | Deconstructing Discourses in Assessments of Child Neglect. | 2021 | England | 1. Semi-structured Interviews 2. Case files   Analysed using narrative enquiry. | | | 1. 1) 2. 8 social workers, 2 social work managers , 8 Children’s Centre workers 3. 2)   5 Child in need, 5 Child Protection | | | To explore discourses in social workers’ assessments of child neglect. | | | | | | A= M, B = H  C = H, D = H  Snowballing sampling method which may not ensure maximum diversity. Explicit theoretical influences and method of analysis. | | Workers felt that thresholds could be too high, meaning that situations got worse. Practitioners felt that the thresholds could be influenced by serious case reviews and resources. Performance data was based on compliance with deadlines rather than quality of assessments. | |  |
|  | Chaudhry, M. N. | The Identification of Child Neglect in Social Work practice. | 2016 | England | Survey analysed using counts, Chi squared. | | | 60 (0.4% response rate) | | | To better understand how social workers identify that neglect has occurred. | | | | | | A = M, B = H  C = M, D = M  Very low response rate. Explored the perceived rather than actual impact of stress on practice. | | 32% agreed with the statement that stress was impacting on their ability to do their job. 45% agreed with the statement that their workload made it hard to follow up on all aspects of cases that they were concerned about. | |  |
|  | Coohey, C. | Making judgments about risk in substantiated cases of supervisory neglect | 2003 | USA | Case control study  Statistical analysis of administrative records using chi squared and t tests followed by logistic regression | | | 602 incidents of substantiated maltreatment of which 158 met criteria | | | To understand how the assessment of risk in supervisory neglect is influenced by the potential harm, parental characteristic and investigatory characteristics | | | | | | A = M, B = H, C = M, D = M  No confidence intervals or sample size calculation. Only a couple of investigator variables were measured. Most of the variables related to the incident or the family. | | The investigator’s level of experience and supervision arrangements were not associated with decisions to a statistically significant degree | |  |
|  | Cowley, L. E. ; Bennett, V. C.; Quinn‐Scoggins, H. D. ; Nuttall, D. ; Wilkins, D. ; Kemp, A. M. | Factors Influencing Clinicians', Health Visitors' and Social Workers' Professional Judgements, Decision-Making and Multidisciplinary Collaboration When Safeguarding Children with Burn Injuries: A Qualitative Study | 2024 | England and Wales | Semi-structured interviews, analysed using a codebook approach to thematic analysis | | | 36 practitioners, of whom 16 were social workers | | | To understand factors influencing practitioners’ judgements and decision making in relation to paediatric burn injuries, which are sometimes caused by neglect | | | | | | A = M, B = H, C = M, D = M  Used a mixture of purposive and snowball sampling. The authors acknowledge that they could not know if thematic saturation was achieved with their sample. Evidence base for questions in interview schedule. Only considers a small part of the social worker’s context (knowledge / training) | | Social workers spoke about a reliance on health professionals to determine how the injury occurred and admitted that they lacked the expertise and had not had sufficient training. They also spoke about the difficulty of getting to speak to doctors as they were so busy | |  |
|  | Craft, J.L. and Staudt, M.M. | Reporting and founding of child neglect in urban and rural communities. | 1991 | USA | Survey asking workers to respond to statements about a vignette using a Likert scale. | | | 25 social workers | | | To perceptions of neglect between urban and rural areas. | | | | | | A = M, B = H  C = M, D = M  Small sample. The authors don’t explain perceived resource differences between urban and rural areas. | | Social workers thought that in urban communities, thresholds would be lower and that cases more likely to be substantiated due to different worker characteristics, availability of services and caseloads. | |  |
|  | Daniel, B., C. Burgess, E. Whitfield, D. Derbyshire and J. Taylor | Noticing and Helping Neglected Children: Messages from Action on Neglect. | 2014 | UK | Focus groups.  Analysis method not specified. | | | 12 meetings with average of 9 attendees from a range of professions. | | | To understand how neglected children are currently helped and how to improve practice. | | | | | | A= M, B = M  C = M, D = M  No mention of sampling strategy or analytical techniques used. Does not differentiate between social workers and other professions. | | Practitioners (from all backgrounds, not just social worker) felt overwhelmed by the broad spectrum of potential neglect. They felt that if they intervened in all cases of neglect, they would overwhelm the system. | |  |
|  | Denne, E., Stevenson, M. and Petty, T. | Understanding how social worker compassion fatigue and years of experience shape custodial decisions | 2019 | USA | Survey asking workers to respond to statements about a vignette about neglect using a Likert scale, analysed using regression analysis. | | | Study 1: 175 of whom 134 social workers recruited online  Study 2: 119 of whom 108 were social workers | | | To understand how compassion fatigue influences children’s social workers’ decision-making | | | | | | A = H, B= H, C= H, D = H  Detailed analysis of the association between different variables. Important topic for this paper. Online recruitment so possibly not a representative sample of social workers but the implications of this are discussed in limitations. | | Compassion fatigue significantly mediated the relationship between the social worker’s number of years of experience and lower standards about what it takes to be a good parent and hopelessness about the child’s prospect of being helped. Compassion fatigue was significantly associated with increased psychological detachment from the child and job efficacy cynicism towards the case which act as mediators. | |  |
|  | Dickens, J., Cook, L. Cossar, J., Okpokiri, C., Taylor, J. and Garstang, J. | Re-envisaging professional curiosity and challenge: Messages for child protection practice from reviews of serious cases in England | 2023 | England | Mixed methods  Statistical analysis of data from reports  Qualitative analysis of a sample of cases and focus group discussions with social workers and managers | | | Study 1: 166, of which 49 selected for in depth analysis  Study 2: 9 reviews and other relevant publications  Studies 3 and 4: 117 | | | To better understand issues of professional curiosity and challenge which feature in many serious case reviews | | | | | | A = H, B = M, C = M, D = M  Serious case reviews are not representative of practice with families where there are concerns about neglect. | | Focus group participants identified heavy workloads, high staff turnover and sickness rates as barriers to good practice. There were also noted to be large numbers of inexperienced, newly qualified staff who might be less confidence about what they should be doing | |  |
|  | Dickens, J. | Child Neglect and the Law: Catapults, Thresholds and Delay. | 2007 | England | Interviews analysed using grounded theory. | | | 23 social workers23 local authority lawyers 6 social work managers, 2 legal managers. | | | To better understand how the legal context influences decision-making concerning neglect. | | | | | | A = H, B = H  C = H, D = H  Sampled from a range of local authorities to capture potential diversity. Staff self-selected cases to talk about . | | In all but one case, legal action followed a “catapult” event. This helped social workers satisfy themselves, their manager, and the court that the threshold was met. There was a risk that the absence of a catapult could result in delay in cases of chronic neglect. | |  |
|  | Doherty, P. | Child protection threshold talk and ambivalent case formulations in ‘borderline’ care proceedings cases | 2017 | England | Ethnographic study: interviews, focus groups, direct observations of practice | | | 47 cases, of which 34 (72%) concerned neglect | | | To understand why cases are categorised as being on the margin of care proceedings, how decisions are resolved and what consideration are critical in resolving them. | | | | | | A = H, B = H, C= H, D = H  Explicit method of analysis. A variety of data sources. No evidence of purposive sampling. | | A social worker described thresholds changing due to management, serious incidents, the media, leading to a move towards defensive practice.  A manager described how a critical incident precipitated court action as, without this, they would have struggled to justify intervening at that particular moment. | |  |
|  | Farmer, E. and Lutman, E. | Working Effectively with Neglected Children and Their Families - What Needs To Change? | 2014 | UK | Case files and interviews analysed using narrative methods. | | | Case files: 138 children  Interviews: 36 social workers . | | | To explore outcomes for neglected children who had been reunited with their parents .after a period in care | | | | | | A = M, B = H  C = M, D = M  Unclear method of analysis. It is not always obvious which findings came from which source.  Sample population was of a small proportion of all neglected children. | | 1/3 of cases had specific incident as trigger for care proceedings.  There was evidence of a failure to intervene in cases of chronic neglect. | |  |
|  | Freisthler, B., Kepple, N. J., Wolf, J. P., Curry, S. R. and Gregoire, T. | Substance use behaviours by parents and the decision to substantiate child physical abuse and neglect by caseworkers. | 2017 | USA | Survey asking workers to respond to statements about a case vignette using a Likert scale. Analysed using T-tests. | | | 721 responses, 467 answered questions in full. | | | To better understand which substances misused by parents are considered by social workers to cause the most harm to children. | | | | | | A = H, B = H  C = M, D = H  Response rates unknown. No sample size calculation but larger sample. It is unknown how respondents compare to non-respondents. Report confidence intervals and non-significant results. | | The following groups of social workers were more likely to substantiate the allegation as neglect:   - Asian/ Pacific Island ethnicity. - With less than a bachelor’s degree. - Not currently working at a child welfare agency. | |  |
|  | Grégoire-Labrecque. G., Lafantaisie, V. ^b^, Trocmé. N., Lacharité. C., Li. P., Audet. G., Sullivan. R/ and Ruiz-Casares, M. | ‘Are We Talking as Professionals or as Parents?’ Complementary views on supervisory neglect among professionals working with families in Quebec, Canada | 2020 | Canada | Focus group discussions asking for their views in general and responses to case vignettes | | | 67 service providers, of whom 16 health and social service providers and 24 youth protection professionals | | |  | | | | | | A = M, B = M, C= M, D = M  Small, convenience sample. Did not collect demographic information about participants. The study recruited professionals from other disciplines as well as child welfare workers and does not differentiate between them when reporting findings. | | Personal and professional experiences influence practitioners’ understandings of supervisory neglect. This includes both experiences of being parented and of parenting their own children. | |  |
|  | Haworth, S., Schaub, J. and Montgomery, P. | Exploring social workers' views on assessing child neglect in England and Wales | 2024 | England and Wales | Survey asking social workers to rate their agreement with various statements via a Likert scale. | | | 129 registered children and families social workers | | | To better understand social workers’ views about assessing child neglect | | | | | | A = M, B = M, C= M, D = M.  Relatively small and non-representative sample of respondents. Only reports the percentage agreeing with certain statements, does not explore associations between answers. Only brief attention to contextual factors. | | Practitioners felt relatively confident in undertaking assessments about neglect and felt that their assessments took consideration of social disadvantage. However, they were less confident in the accuracy of their assessments or that they were informed by research evidence.  When asked what would help give them more time to undertake assessments into neglect, 80.3% listed lower caseloads, 58.6% better multi agency working, 53.5% less bureaucracy, 49.6% better organisational support for direct work with families and 48.8 shorter and more concise assessment forms | |  |
|  | Hood, R., Gorin, S., Goldacre, A., Muleya, W., Bywaters, P. | Exploring drivers of demand for child protection services in an English local authority | 2020 | England | Focus groups and interviews with practitioners and managers from statutory Children’s Services and Early Help, analysed using thematic analysis. | | | 25 | | | To understand the reasons for a rise in demand for child protection services in an English local authority. | | | | | | A = H, B= M, C = M, D = M  Purposive sample. The questions asked about reasons for rising demand for interventions for maltreatment generally and only briefly reports findings specific to neglect. | | Some participants suggested that rising numbers of strategy discussions and child protection conferences were a response to a recent Ofsted inspection which had focused on neglect. They also reported that management had adopted a more interventionist culture, meaning that action would be taken on neglect cases that would previously have drifted | |  |
|  | Horwath, J. | Child neglect is my view your view? Working with cases of child neglect in the North Eastern Health Board | 2001 | Ireland | Content analysis of case files, questionnaire, focus groups. | | | 57 cases (137 children)  40 questionnaire responses  5 focus groups (25 practitioners, 9 managers). | | | The study was commissioned by the Health Board to explore the implementation of the 1999 Child First Guidelines. | | | | | | A = H, B = B  C = H, D = H  Not peer reviewed. 53% response rate (but 12% off sick). Mixed methods to give a range of perspectives. Small sample but appropriate sampling techniques. Coding frame developed in pilot study. | | Practitioners felt that there was a level of subjectivity in decision making. They felt that different managers had different thresholds and that this influenced decision making. Practitioners also felt that resources and time constraints influenced decisions. | |  |
|  | Horwath, J | Is this child neglect? The influence of differences in perceptions of child neglect on social work practice. | | | | 2004 | This paper is based on the study discussed in Horwath 2001. | | | | | | | | | | A = H, B = H  C = H, D = H  Peer reviewed. As above. | | Better resourced teams looked at a wider range of risk factors and adopted a lower threshold for intervention. 7 out of 9 managers agreed that “the criteria for triggering child protection procedures can vary depending on which manager is involved”. | |  |
|  | Horwath, J. | Identifying and assessing cases of child neglect: learning from the Irish experience. | | | | 2005 | This paper is based on the study discussed in Horwath 2001. | | | | | | | | | | A = H, B = H  C = H, D = H  Peer reviewed. As above. | | Workload and stress influenced assessments. There were differences in practice between different teams. | |  |
|  | Horwath, J. and Tarr, S. | Child Visibility in Cases of Chronic Neglect: Implications for Social Work Practice. | 2015 | England | Qualitative  Content analysis of   1. case files 2. interviews 3. focus groups.   Quantitative   1. Survey | | | 1) 21 case files  2) 18 interviewees  3) 4 focus groups containing 34 professionals   1. 4) 162 | | | To better understand why a lack of focus on the child has been identified in previous work. | | | | | | A= H, B= H  C = H, D = H  Good synthesis of findings from different methods. Appropriate sampling methods (purposive) and method of analysis (thematic content analysis). Response rate for survey unclear. Only 32% of respondents were social workers. | | Workers often looked for "tangible and easily measurable actions" taken by family or professional when assessing progress e.g. keeping appointments, having food in the cupboard, school attendance. It was assumed but not demonstrated that this led to better outcomes for the children. | |  |
|  | Janczewski, C.E. | The influence of differential response on decision-making in child protective service agencies. | 2014 | USA | Multivariate regression analysis of administrative data. | | | 994,045 neglect investigation records from 2010 data set (Oct 2009 to Sep 2010) | | | To better understand how differential response influences outcomes. | | | | | | A = H, B = H  C = H, D = H  Data cleaning undertaken to address systematic biases in dataset. Controlled for potential confounding factors in analysis e.g. poverty rates. | | Counties implementing differential response have lower investigation and substantiation rates. Amongst investigated cases there are higher substantiation rates. | |  |
|  | Janczewski, C. E. and Mersky, J. P. | What's so different about differential response? A multilevel and longitudinal analysis of child neglect investigations | 2016 | USA | Multivariate analysis of cross-sectional data from administrative records.  Longitudinal analysis using a mixed-effect time series approach to test whether differences in case flow between differential response and non-differential response areas corresponds with the launch of differential response. | | | 997,512 cases from 269 counties  Cross sectional analysis uses 2010 data  (data appears to overlap with that used in above study)  Longitudinal analysis uses data 2001-2010 | | | To better understand how differential response influences investigation and substantiation decisions and its possible influence on predictors of maltreatment and CPS involvement | | | | | | A = H, B = H, C = H, D = H  Detailed statistical analysis considering the interaction of different variables | | In counties using differential response, neglect reports were 2.17 times more likely to be substantiated compared to counties not using DR. The influence of having a prior report was also greater. Differences in substantiation rates between areas predated the introduction of DR | |  |
|  | King, B., Black, T., Fallon, B. and Lung, Y. | The role of risk in child welfare decision-making: A prospective cohort examination of families transferred to ongoing child protection services after an investigation. | 2021 | Canada | Prospective cohort study . | | | 34,397 families | | | To better understand factors which predict referral for ongoing services following initial report, and the role of risk within this. | | | | | | A = M, B = H  C = M, D = M  The study is well designed and conducted. However the authors were a little unclear about what ongoing services they were measuring, which made the findings hard to interpret for a non-Canadian reader. | | In Ontario, cases featuring neglect were more likely to be referred for ongoing support than physical or sexual abuse. The authors suggest that this is due to the use of an assessment tool which captures the risk of future harm. Rates of transfer between agency varied from just under 15% to 35%. Rural agencies were more likely to offer a service as there were fewer alternatives. | |  |
|  | Lee, T. | Catching a Case: Inequality and fear in New York City’s child welfare system | 2016 | USA | Survey of parents  Interviews  Informal conversations  Observations of court rooms, parenting classes and support groups  Conferences | | | 28 interviews with lawyers, judges and caseworkers | | | To understand state practice that shape the lives of women of colour in poverty to understand how they reproduce inequalities | | | | | | A = L, B = M, C = M , D = M  Sampling strategy and method of analysis are unclear. Mainly looks at court hearings which are not representative of practice as a whole. No discussion of competing voices in the data (e.g. parents v professionals) or reflexivity, despite the author clearly having some strong preconceptions. | | The legal definition of neglect encouraged practitioners to look at problems in isolation from their social context.  The study found that “rather than providing oversight…. family court too often rubber-stamps caseworkers’ judgements with little scrutiny” [140], allowing “exaggerated claims” [171] to go unchallenged.  The author suggests a number of reasons for this. These include the courts being too busy to have full hearings, judges having a conflict of interest due to being appointed by the mayor (part of the city administration) and attorneys being poorly paid and overworked so not having the time or interest to fight for families, detached from emotions of actual removal.  Caseworkers and judges feared being responsible for a child death and this led them to make more cautious decisions: “Cover Your Ass” moves [59]. This was heightened after a high-profile child death in the media.  Caseloads for both case workers and the courts were high leading to an emphasis on completing steps, rather than making adequate decisions. | |  |
|  | Proctor, S.N. | Implicit bias, attributions, and emotions in decisions about parents with intellectual disabilities by child protection workers. | 2011 | USA | Survey asking participants to respond to case vignettes. | | | 230 | | | To understand if stereotyping affects workers’ judgements about parents with learning disabilities. | | | | | | A = M, B = H  C = M, D = M  The response rate to postal surveys is very low (8%) or unknown. Non-response bias unknown. No sample size calculation. Confidence intervals and non-statistically significant results not provided. Considers potential interaction between variables. | | Social workers’ age was significantly associated with their perception of risk of neglect when assessing parents with intellectual disabilities, and with their implicit stereotypes, implicit attitudes, and anger. | |  |
|  | Rodrigues, L., Calheiros, M., Pereira, C. | The decision of out-of-home placement in residential care after parental neglect: Empirically testing a psychosocial model | 2015 | Portugal | Survey asking participants to respond to case vignettes and also answer questions about their beliefs, attitudes and emotions relating to the decision using a Likert scale. Structured equation modelling | | | 195 workers from 150 units | | | To test a psychological model of decision-making about placing children in out of home care. | | | | | | A = M, B = H, C = H, D = H  Low participation rate (15.5%) but subsequent analysis suggested that the sample size was adequate. | | Workers decisions about whether or not to recommend residential care for a neglected child were determined by cognitive, social, affective, value-laden and experience variables and the perceived risk. | |  |
|  | Rose , S. J. and Meezan, W. | Child neglect: A study of the perceptions of mothers and child welfare workers. | 1995 | USA | Survey asking participants to respond to case vignettes. | | | 49 protective service investigators.  74 protective service caseworkers | | | To better understand views about neglect thresholds. | | | | | | A = M, B = H  C = M, D = M  Small sample and no sample size calculation. Workers recruited from office, minimising non-response bias. Only reports statistically significant results. | | Social workers in investigative roles rated incidents as significantly more serious than did workers in service roles. The authors suggest that this may be due to differences in their professional background and differences in nature of the role. | |  |
|  | Rose , S. J. and Meezan, W. | Variations in perceptions of child neglect. | | | | | | | 1996 | | | | This paper was based on the same study as Rose and Meezan 1995 and reports the same findings. | | | | | | | |  |
|  | Rossi, P. H., Schueran, J., and Budde, S. | Understanding decisions about child maltreatment. | 1999 | USA | Multinomial regression analysis and Kish design effect measures to analyse results of survey asking workers to respond to statements about a case vignette using a Likert scale. | | | 27 "experts" i.e. middle managers or academics.  103 social workers. | | | To better understand how decisions are made in child protection. | | | | | | A = M, B = H  C = M, D = M  Some sampling random, also asked for volunteers, where there is a higher risk of sampling bias. Fair response rates of 73% and 89%. Relatively small sample and no sample size calculation. Confidence intervals not stated. Original vignettes not provided. | | Worker characteristics had less influence on decisions than case characteristics. “Experts” were more likely than workers to make use of preservation services. For serious matters where parents were willing to engage, respondents were less likely to recommend that a neglected child be taken into custody if they were told that family preservation services were available. | |  |
|  | Scourfield, J. | The rediscovery of child neglect. | 2000 | England | Ethnography analysed using grounded theory.   1. Interviews 2. Case files 3. Observation of office environment, not client interactions. | | | 1 social work office (number of workers not specified) | | | To explore the influence of 2 competing discourses on perceptions of neglect. | | | | | | A= H, B = H  C = H, D = H  Explicit method of analysis. Claims appear supported by evidence presented. Only observed practice in an office, not with clients, can only shed light on how neglect constructed in discourse between social workers. | | Social workers were most influenced by the emphasis on physical care needs from Bridge Childcare Consultancy’s report into the death of baby “Paul”. Scourfield suggests that this is perhaps partly because of the “usefulness of the observable body as evidence…concrete proof” (p377-8). They were aware of the emphasis on emotional care in Dept for Health’s *Child Protection: Messages from Research*. | |  |
|  | Segal, U.A. | Child abuse in India: An empirical report on perceptions . | 1992 | India | Survey asking participants to respond to case vignettes. | | | 45 social workers in India | | | To understand attitudes towards child abuse in India and compare these with data about social workers from the USA from a different study (Giovannoni, 1979). | | | | | | A = M, B = H  C = M, D = M  Relatively small sample. 60% response rate. Non-random sampling, creating risk of sampling bias. No sample size calculation. No confidence intervals provided.  No information given about the USA data from the Giovannoni study. Quite an old study now and India has undergone significant cultural change in this time. | | Social workers in the USA rated following as more serious than social workers in India did: medical neglect, nutritional neglect. Indian social workers rated the following as more serious than US workers: alcohol/drug abuse, cleanliness, and housing. | |  |
|  | Taylor, J., Dickens, J., Garstang, J., Cook, L., Hallett, N. and Molloy, E. | Tackling the ‘normalisation of neglect’: Messages from child protection reviews in England | 2024 | England | Qualitative analysis (reflexive thematic analysis) of serious case reviews concerning neglect 2017-2019. | | | 166, of which 12 analysed in depth | | | To identify areas for improvement in keeping children experiencing neglect safe. | | | | | | A = H, B = M, C = M, D = M.  Serious case reviews are not representative of practice with families where there are concerns about neglect. | | There is a risk that neglect may become normalised for experienced practitioners, meaning that they do not take action. It may also be so overwhelming to deal with that it distracts practitioners from other forms of maltreatment | |  |
|  | Tufford, L., Bogo, M. & Asakura, K. | How Do Social Workers Respond to Potential Child Neglect? | 2015 | Canada | Objective Structural Clinical Examination followed by qualitative analysis of reflective dialogues . | | | 23 student social workers | | | To explore how early and mid-career social workers, who are mandated reporters, recognise and respond to child neglect. | | | | | | A = H, B = M  C = M, D = M  2 researchers coded data.  Sample is of social work students, rather than experienced practitioners so may not be generalisable. Only 1 case study | | In reflective dialogues, social workers drew parallels between their own situation and the mother’s e.g. their age, immigrant status in making sense of the neglect allegation. Some participants appeared overwhelmed by the enormity of the mother’s needs and could not make an objective assessment of risk. | |  |
|  |  |  |  |  |  |  |  |  |  |  | |  | |  | |  | |  | |  | |
