# Supplementary material for: Which Factors in a Child Welfare Worker’s Environment Influence Their Decision-Making About Neglect? A Systematic Narrative Literature Review
Source: Trauma Violence Abuse. 2025 Mar 12;27(3):611–23. doi: 10.1177/15248380251320987 (PMC13291384; doi:10.1177/15248380251320987)
Supplement: sj-docx-5-tva-10.1177_15248380251320987 – Supplemental material for Which Factors in a Child Welfare Worker’s Environment Influence Their Decision-Making About Neglect? A Systematic Narrative Literature Review [file sj-docx-5-tva-10.1177_15248380251320987.docx]

Appendix 5: Table 3:

How Popay et al’s method for narrative synthesis

was adapted and used for the purpose of this review.

| Analytic method | Explanation of how followed in this review’s synthesis |
| --- | --- |
| Tabulation  To organise results, compare key characteristics and identify patterns | Both quantitative and qualitative studies were entered onto a spreadsheet.  Columns were created for the key characteristics of each study, such as the country in which the study was conducted, the sample size and sample characteristics. This was helpful in identifying the variety in the studies. Columns were also added inductively for research questions emerging in the literature, to allow for comparison of these issues between studies. The column headings were differences between social workers, the prioritisation of neglect compared to other forms of abuse, the prioritisation of different neglect subtypes, perceived risk factors for neglect, the relationship between neglect and poverty and how perceptions varied in relation to minority groups. |
| Translation of quantitative data into themes | A textual description was created of the findings of each quantitative paper, and these were entered into the appropriate columns of the spreadsheet, alongside findings from qualitative studies, to enable comparison |
| Assessing the robustness of studies | Gough’s weight of evidence tool (Gough, 2007) allowed consideration of the relevance of the study to the questions in this review, as well as the quality of the paper in its own right. This was chosen given that many of the studies selected asked a slightly different question to this review.  The Mixed Methods Appraisal Tool (Hong QN & M-P, 2018) was used to help answer question A in Gough’s model, the quality of the evidence in its own right. |
| Grouping | Two different types of evidence were identified:   1. What child welfare workers thought would influence their decisions in hypothetical situations 2. What factors affected decision-making in practice |
| Translating data: thematic analysis | Thematic analysis was used to identify the main themes emerging in the literature, taking as a starting point the research questions tabulated, as described above.  Themes included:   - factors relating to the individual social worker. - resource availability - national policy - the family courts |
| Methodological triangulation | Where findings varied between studies, the methodologies were examined to see to what extent these might explain the differences. Several were discussed with the wider research team. |
| External validation | Drafts of this manuscript were discussed with several groups of stakeholders to see if the narrative was credible and consistent with their experiences. These groups included:   - A team meeting of experienced hospital social workers, all of whom had previous experience working in the local authority. - A group of experts by experience in children’s social care - Academics working in the field of child development and mental health |
